# Rapid nectar-meal effects on a predator's capacity to kill mosquitoes

**DOI:** 10.1098/rsos.140426

**Published:** 2015-05-13

**Authors:** Georgina E. Carvell, Josiah O. Kuja, Robert R. Jackson

**Affiliations:** 1 School of Biological Sciences, University of Canterbury, Private Bag 4800, Christchurch, New Zealand; 2 International Centre of Insect Physiology and Ecology (ICIPE), Thomas Odhiambo Campus, PO Box 30, Mbita Point 40305, Kenya; 3 Department of Biological Sciences, Jomo Kenyatta University of Agriculture and Technology, Nairobi 00200, Kenya

**Keywords:** nectarivory, Salticidae, *Evarcha culicivora*, *Lantana camara*, *Ricinus communis*, *Parthenium hysterophorus*

## Abstract

Using *Evarcha culicivora*, an East African jumping spider (Salticidae), we investigate how nectar meals function in concert with predation specifically at the juvenile stage between emerging from the egg sac and the first encounter with prey. Using plants and using artificial nectar consisting of sugar alone or sugar plus amino acids, we show that the plant species (*Lantana camara*, *Ricinus communis*, *Parthenium hysterophorus*), the particular sugars in the artificial nectar (sucrose, fructose, glucose, maltose), the concentration of sugar (20%, 5%, 1%) and the duration of pre-feeding fasts (3 days, 6 days) influence the spider's prey-capture proficiency on the next day after the nectar meal. However, there were no significant effects of amino acids. Our findings suggest that benefits from nectar feeding are derived primarily from access to particular sugars, with fructose and sucrose being the most beneficial, glucose being intermediate and maltose being no better than a water-only control.

## 2. Introduction

There has been a longstanding interest in explaining the origins and adaptive significance of omnivory (i.e. feeding at more than one trophic level). Frequently considered hypotheses include minimizing overexposure to toxins associated with otherwise superior food and surviving periods when superior food is scarce by relying on inferior food sources [1–3]. Omnivory is especially interesting when animals traditionally envisaged as being simply predators are shown also to take nutrients directly from plants [4]. For example, spiders are widely regarded as being exclusively predators, but *Bagheera kiplingi* is a striking exception [5]. This Central American jumping spider (Salticidae) cohabits with ants (*Pseudomyrmex* spp.) on ant-acacias (*Vachellia* spp.), where it sometimes captures and eats the ants, but it feeds primarily from the Beltian bodies (i.e. specialized leaf tips) that also serve as the ants' primary food [6]. No other spiders are known to express a comparable level of herbivory, but many spiders are now known to supplement a primarily predatory diet with plant products, including pollen [7–12], honeydew [13–15] and especially nectar taken from flowers or extra-floral nectaries (EFNs) [16–22]. For two non-salticid spiders [23,24], *Cheiracanthium mildei* (Miturgidae) and *Hibana velox* (Anyphaenidae), experiments have shown that nectarivory, when combined with feeding as a predator, improves survival, growth and fecundity.

Our research is different because we use a salticid spider and we investigate a more rapidly expressed nectar-derived benefit. Owing to the exceptional spatial acuity of their large, complex principal eyes [25,26], salticids can readily detect and identify prey from a distance and, for some salticid species, there is experimental evidence of highly specific vision-based prey-choice decisions [27]. However, the level of specificity expressed by *Evarcha culicivora*, the species we consider here, is remarkable even by salticid standards [28]. This East African salticid feeds indirectly on vertebrate blood by actively choosing blood-carrying female mosquitoes as preferred prey [29], and *E. culicivora* chooses species from the genus *Anopheles* as its preferred mosquitoes [30,31]. From a human perspective, a preference for *Anopheles* is particularly relevant because all human malaria vectors belong to this genus [32].

Besides having an exceptional capacity for seeing detail, many salticids also make extensive use of chemoreception, including olfaction (e.g. [33]). The role of olfaction in the biology of *E. culicivora* is especially complex [34–38] and includes odour-mediated responses to plants [39]. In the field, *E. culicivora* is frequently found on *Lantana camara* and *Ricinus communis* [40], two of the most common plant species in its habitat [41–43] and *E. culicivora* is attracted to the odour of both of these plant species in olfactometer experiments [39].

Nectar meals may be especially important for the early instar juveniles of *E. culicivora*. *β*-caryophyllene and *α*-humulene, the dominant sesquiterpenes from the headspace of *L. camara*, attract the adults and the juveniles of *E. culicivora* [44], but pre-trial fasts make the early instar juveniles, but not the adults, of *E. culicivora* more strongly predisposed to move towards a source of these volatile compounds [45]. Evidence from cold-anthrone testing also shows that *E. culicivora* ingests nectar from *L. camara* and other plants, but the early instar juveniles of *E. culicivora* appear to feed on nectar considerably more often than adults [46].

The hypothesis we consider here is that nectar meals make early instar juveniles more proficient at capturing mosquitoes. Part of the rationale for this hypothesis is that mosquitoes are much larger than the early instar juveniles of *E. culicivora*. After being attacked, mosquitoes sometimes shake off the early instar individuals, but larger individuals of *E. culicivora* appear to have no difficulty holding on [47].

We also consider whether prey-capture proficiency is affected by the plant species from which the nectar is derived, the dilution of sugar in solution, the particular sugars acquired by the spider or the amino acid content of nectar. We based our choice of sugars, amino acids and concentrations on current understanding of nectar chemistry. A few exceptions notwithstanding, nectar is primarily a sugar solution, with amino acids being the second most common component. Total sugar concentration varies, but around 20–40% solute is typical, with amino acid concentration tending to be closer to 1%. Sucrose, glucose and fructose are usually the dominant nectar sugars and other sugars, when present, are usually found at considerably smaller concentration [48,49].

## 3. Material and methods

### 3.1 General

Females of *E. culicivora* put their eggs in silk egg sacs situated inside cocoon-like nests and, after hatching, the juveniles leave the nest at roughly the same time and spread about in the cage [50]. Here, we reserve the expression ‘juvenile’ for these active newly emerged spiders. The juveniles that we used were second- and third-generation individuals from laboratory cultures derived from individuals collected at our field site in Mbita Point, Western Kenya (elevation 1200 m a.s.l.; latitude 0°30′ S; longitude 34°10′ E). As our basic methods for rearing, maintaining and testing spiders were as in earlier studies (e.g. [37]), only essential details are stated here.

We isolated juveniles on the day of emergence, put them into separate maintenance cages (cylindrical, height 55 mm, diameter 45 mm, made from clear plastic) and then kept them without food for a specified pre-feeding interval. Each spider had continual access to water via a cotton roll (‘dental wick’) inserted into a hole in the bottom of the cage and positioned so that it extended into a water-filled plastic pot below the cage. There was a mesh-covered hole in the top of the cage for ventilation. A second hole in the top of the cage was plugged with a rubber stopper which could be removed when introducing prey. All holes were 8 mm in diameter.

We assigned spiders at random to one of 18 meal-type groups and, after a fast of a specified duration, we gave the spider access to a first meal corresponding to the meal-type group (table 1). The first meal was artificial nectar (i.e. a solution of sugar or sugar plus amino acids), a plant or a water-only control (table 1). For the control and all artificial nectar, we used distilled water. For plants, we used *L. camara* and *R. communis*, as well as *Parthenium hysterophorus*, a species that is common in the same habitat but not known to attract *E. culicivora* in olfactometer experiments (R. R. Jackson 2008, unpublished data).

**Table 1.** Meal-type descriptions and logistic regression results for 18 meal-type groups. (H_2_O, *n*=200. All other groups, *n*=50.)

abbreviation	meal-type group	coefficient	s.e.	*z*	*p*-value
LC-C	*Lantana camara* cutting	3.07	0.36	8.57	<0.001
RC-C	*Ricinus communis* cutting	1.91	0.27	6.99	<0.001
PH-C	*Parthenium hysterophorus* cutting	1.06	0.25	4.23	<0.001
LC-SAA	full artificial nectar of *L. camara*	2.71	0.32	8.39	<0.001
LC-S	sugar-only artificial nectar of *L. camara*	2.97	0.35	8.54	<0.001
Suc-20	sucrose-only artificial nectar at high concentration (20%)	2.79	0.33	8.45	<0.001
Suc-5	sucrose-only artificial nectar at medium concentration (5%)	1.91	0.27	6.99	<0.001
Suc-1	sucrose-only artificial nectar at low concentration (1%)	0.61	0.25	2.43	0.015
Fru-20	fructose-only artificial nectar at high concentration (20%)	2.49	0.31	8.14	<0.001
Fru-5	fructose-only artificial nectar at medium concentration (5%)	2.06	0.28	7.37	<0.001
Fru-1	fructose-only artificial nectar at low concentration (1%)	0.53	0.25	2.09	0.037
Glu-20	glucose-only artificial nectar at high concentration (20%)	1.49	0.26	5.75	<0.001
Glu-5	glucose-only artificial nectar at medium concentration (5%)	1.06	0.25	4.23	<0.001
Glu-1	glucose-only artificial nectar at low concentration (1%)	0.40	0.25	1.56	0.119
Mal-20	maltose-only artificial nectar at high concentration (20%)	0.82	0.25	3.27	0.001
Mal-5	maltose-only artificial nectar at medium concentration (5%)	0.65	0.25	2.60	0.009
Mal-1	maltose-only artificial nectar at low concentration (1%)	0.35	0.25	1.38	0.167
H_2_O	control (distilled water alone)	0.37	0.18	2.11	0.035

For the water-only control group, the individuals used were derived from 29 sibships. For each other group, the individuals used were derived from 8–10 sibships. A ‘sibship’ is defined as the progeny of a particular male and female. No sibships contributed individuals to more than one group. The number of individuals from any one of the sibships was never more than eight or less than three. Owing to the way we took individuals from a range of sibships, we did not include sibship as a variable in our data analyses.

The sugar and amino acid content of *L. camara* nectar is known (cited in [51] as personal communication from Irene Baker to Alm *et al.*): sucrose 187.25 g l^−1^, fructose 57.00 g l^−1^, glucose 55.80 g l^−1^, proline 0.256 g l^−1^, glycine 0.178 g l^−1^, serine 0.144 g l^−1^, glutamine 0.136 g l^−1^, threonine 0.080 g l^−1^, alanine 0.064 g l^−1^, asparagine 0.056 g l^−1^, tyrosine 0.040 g l^−1^, glutamic acid 0.048 g l^−1^, arginine 0.032 g l^−1^ and valine 0.016 g l^−1^. For our experiments, we made two artificial nectar blends based on the reported ratio of the three sugars and the four dominant amino acids in this plant's nectar.

Full artificial *L. camara* nectar: sucrose 187.3 g l^−1^, fructose 57.0 g l^−1^, glucose 55.8 g l^−1^, proline 0.3 g l^−1^, glycine 0.2 g l^−1^, serine 0.1 g l^−1^, glutamine 0.1 g l^−1^.

Sugar-only artificial *L. camara* nectar: sucrose 187.3 g l^−1^, fructose 57.0 g l^−1^, glucose 55.8 g l^−1^.

The sugar content of *R. communis* and *P. hysterophorus* nectar is not known precisely, but the floral tissues of these plants contain sucrose, fructose, glucose and other sugars, including especially maltose [52]. We included maltose in our experiments as a sugar that may be in *R. communis* nectar, but is not known to be present in the nectar of *L. camara* or prevalent in the nectar of plants, in general.

For each meal-type group, there were two fasting-duration subgroups (3 day and 6 day): spiders kept without access to food for 3 days or 6 days before being given access to the meal corresponding to the meal-type group (table 1) on the 4th or 7th day.

### 3.2 Experimental procedure

The spider's first meal was placed in its cage at 8.00 and removed 60 min later (laboratory photoperiod 12 L : 12 D, lights on at 7.00). During this feeding period, the spider was observed continuously. For each plant meal, the plant used in an experiment was a cutting taken from a living plant in the field. In each instance, the plant was held in a closed plastic box under 100% carbon dioxide for 10 min and then examined under a microscope for any arthropods that might have remained (none were found). The cut end of the stem was the only incision or wound on the plant and it remained outside the cage (i.e. the stem, positioned alongside the cotton roll, went through the hole in the bottom of the cage so that the cut end was in the pot of water below the cage). The remainder of the plant (stems, flowers and leaves) was inside, and almost filled, the cage.

When the first meal was artificial nectar or the water-only control, a disc (diameter 5 mm, thickness 2 mm, cut from a clean kitchen sponge) was submerged in the specified solution (or water alone for the control) for 10 s at 7.30 [46]. The sponge disc was then attached by a pin to the centre top of a clean, dry cotton roll. The cotton roll that was providing water to the spider was removed at 8.00 and replaced with the clean cotton roll along with the attached solution-soaked sponge disc. The sponge disc was positioned horizontally at the top of the pin (25 mm above the cage floor, 30 mm below the cage ceiling and 22.5 mm from the side of the cage). During each trial, the cotton roll remained dry (i.e. there was no water in the pot below the cage).

We removed any spider that failed to feed while the first meal was on offer and replaced it with another spider. Our criterion for recording that a spider fed was seeing its mouthparts pressed against a plant (flower petal, leaf or stem), or against a sponge disc that had been soaked in artificial nectar [46].

On the following day at 7.30, each test spider was transferred to a testing cage. Testing cages were similar to maintenance cages, but larger (height 110 mm, diameter 60 mm). The larger size allowed sufficient space for mosquitoes to fly, making them harder for the spider to capture. At 8.00, 24 h after the first meal, we removed the stopper from the hole in the top of the cage and, using an aspirator, introduced four mosquitoes (*Anopheles gambiae s.s*.), after which the stopper was returned to the hole. The mosquitoes were taken from stock cultures and had fed on blood 4 h before being used in the experiments (for methods pertaining to mosquito culturing and feeding, see [53]).

The outcome of a trial was recorded as successful when the test spider attacked the mosquito, held on and then fed and it was recorded as unsuccessful when the spider attacked the mosquito, but failed to hold on and feed. Whenever 2 h elapsed without the test spider attacking a mosquito, the test ended and these spiders were excluded from further analysis (i.e. all data came from instances of a test spider attacking the prey and then being either successful or unsuccessful at capturing the prey; no instances of multiple attacks were considered).

### 3.3 Data analysis

The statistics package R [54] was used for all data analyses. We applied a logistic regression to prey-capture data, with each instance of the spider capturing the prey being coded as 1 and each instance of the spider failing to capture the prey it attacked being coded as 0. Meal type was included as a factor in the model and pre-trial fast duration (3 or 6 days) was included as a standard variable. Using the ‘glm’ function in the stats package, we created logit models and compared them by likelihood-ratio testing from the ‘anova’ function in the stats package. We made pairwise comparisons of coefficients by using Wald tests (based on *χ*^2^) with Holm–Bonferroni corrections from the aod package [55].

## 4. Results

### 4.1 General

The best-fit logistic model was

$$P\;(\mathrm{capture})=\frac{e^{\left(\beta_{m}-0.21d\right)}}{1+e^{\left(\beta_{m}-0.21d\right)}},$$

where *P* (capture) is prey-capture success expressed as the probability that, after making an attack, the spider will hold on and eat the prey, *e* is the base of the natural logarithm, *d* is the pre-trial fast duration in days, −0.21 (*z*=0.03, s.e.=−6.78, *p*<0.001) is the coefficient for fast duration and *β*_*m*_ is the coefficient for meal-type *m* (table 1).

This model was a significantly better predictor of the data than a reduced (intercept only) model (likelihood-ratio testing, *χ*^2^=410.81, *p*<0.001), and it was not significantly different from an expanded model that included interaction terms (*χ*^2^=−11.26, *p*=0.843). There were no significant interaction effects in the expanded model and Akaike's Information Criterion (AIC) was smaller for the best-fit model than for the reduced model (ΔAIC=22.7) or the expanded model (ΔAIC=374.8).

**Figure 1.**
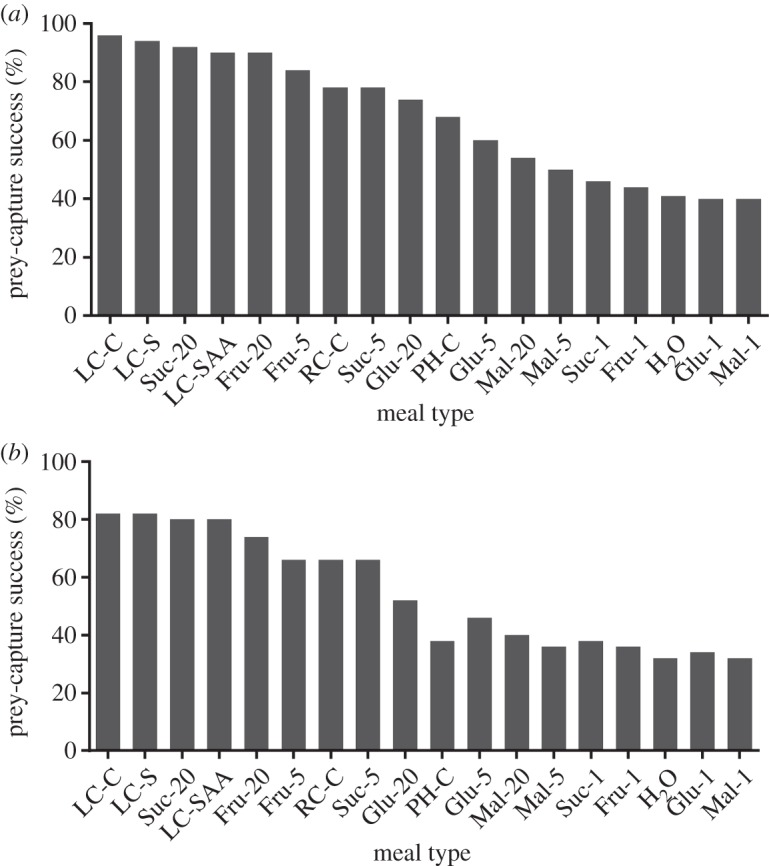
For *Evarcha culicivora* juveniles, percentages of individuals from different meal-type group that, after attacking, succeeded in capturing mosquitoes. Abbreviations for groups defined in table 1. *N*=200 for H_2_O and 50 for each other group, (*a*) 3 day pre-trial fast. (*b*) 6 day pre-trial fast. Sequence of groups on *x*-axis for 3 day and for 6 day fast: from highest to lowest percentage after 3 day fast. Percentages lower for 6 day than for 3 day fasts, but rankings of groups by percentage comparable for 3 day and 6 day fasts.

There was a significant effect of pre-trial fast duration (figure 1; Wald test, $\chi_{1}^{2}=40.8$, *p*<0.001) on the spider's success, with fewer spiders capturing prey after the longer fast (odds ratio=0.81). We also found a significant effect of meal type (Wald tests, $\chi_{18}^{2}=332.4$, *p*<0.001).

### 4.2 Plants and artificial nectar compared with the water-only control

We use the expression ‘effect’ for instances of spiders from a plant group or an artificial nectar group having significantly greater prey-capture success than spiders from the water-only control. We found an effect when spiders fed on each of the three plant species and when spiders fed on artificial *L. camara* nectar (table 2). When we used single-sugar solutions, we found an effect when the spiders fed on 20% and 5% solutions of sucrose, fructose and glucose. However, we found no effect for spiders that fed on 1% solutions of these sugars and no effect even at 5% or 20% when the sugar was maltose.

**Table 2.** Pairwise comparisons (Wald tests based on *χ*^2^) showing effect of each meal type on prey-capture success. (In each instance, coefficient for meal type in first column compared with coefficient for water-only control.)

abbreviation	meal-type group	*χ*^2^	*p*-value
LC-C	*Lantana camara* cutting	63.58	<0.001
RC-C	*Ricinus communis* cutting	38.10	<0.001
PH-C	*Parthenium hysterophorus* cutting	9.13	0.003
LC-SAA	full artificial nectar of *L. camara*	60.23	<0.001
LC-S	sugar-only artificial nectar of *L. camara*	63.09	<0.001
Suc-20	sucrose-only artificial nectar at high concentration (20%)	61.38	<0.001
Fru-20	fructose-only artificial nectar at high concentration (20%)	55.98	<0.001
Glu-20	glucose-only artificial nectar at high concentration (20%)	22.50	<0.001
Mal-20	maltose-only artificial nectar at high concentration (20%)	3.78	0.052
Suc-5	sucrose-only artificial nectar at medium concentration (5%)	38.10	<0.001
Fru-5	fructose-only artificial nectar at medium concentration (5%)	43.65	<0.001
Glu-5	glucose-only artificial nectar at medium concentration (5%)	9.13	0.002
Mal-5	maltose-only artificial nectar at medium concentration (5%)	1.47	0.225
Suc-1	sucrose-only artificial nectar at low concentration (1%)	1.05	0.305
Fru-1	fructose-only artificial nectar at low concentration (1%)	0.43	0.513
Glu-1	glucose-only artificial nectar at low concentration (1%)	0.01	0.925
Mal-1	maltose-only artificial nectar at low concentration (1%)	0.01	0.925

### 4.3 Plants compared

**Figure 2.**
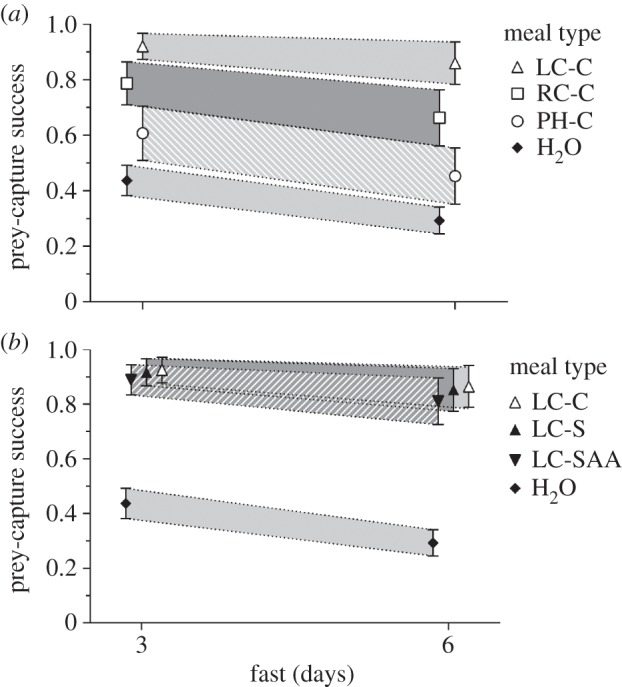
For *Evarcha culicivora* juveniles, predicted prey-capture success (i.e. probability of capture success after attacking mosquito) plus 95% confidence intervals after 3 day and 6 day fast. Predictions derived from logistic model (see text). Abbreviations for groups defined in table 1. (*a*) Spiders that fed on different plant species. (*b*) Spiders that fed on *L. camara* or on artificial *L. camara* nectar.

Prey-capture success was significantly higher when spiders fed on *L. camara* instead of *R. communis* (figure 2*a*; *χ*^2^=8.79, *p*=0.003) or *P. hysterophorus* (*χ*^2^=27.88, *p*<0.001) and also when they fed on *R. communis* instead of *P. hysterophorus* (*χ*^2^=7.75, *p*=0.005).

### 4.4 *Lantana camara* and artificial *Lantana camara* nectar compared

The prey-capture success of spiders that fed on *L. camara* was not significantly different from the success of spiders that fed on either type of artificial *L. camara* nectar (figure 2*b*): full (*χ*^2^=0.71, *p*=0.399), sugar-only (*χ*^2^=0.05, *p*=0.824). Spiders that fed on full and sugar-only artificial *L. camara* nectar were not significantly different from each other (*χ*^2^=0.39, *p*=0.530).

### 4.5 Multiple and single-sugar nectar compared

**Figure 3.**
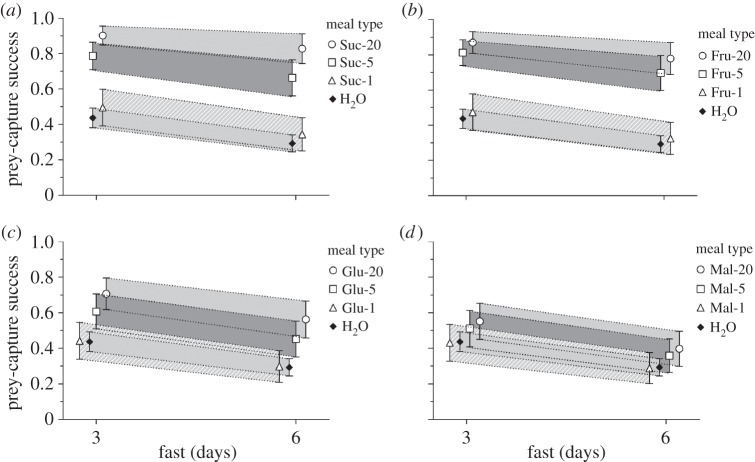
For *Evarcha culicivora* juveniles, predicted prey-capture success (i.e. probability of capture success after attacking mosquito) plus 95% confidence intervals after 3 day and 6 day fast. Predictions derived from logistic model (see text). Abbreviations for groups defined in table 1. Spiders fed on different concentrations of (*a*) sucrose, (*b*) fructose, (*c*) glucose and (*d*) maltose.

Sugar-only artificial *L. camara* nectar (figure 2*b*) was compared with each single-sugar solution (figure 3) at the highest concentration (20%). Prey-capture success was significantly higher after feeding on artificial *L. camara* nectar than after feeding on glucose alone or maltose alone: glucose (*χ*^2^=15.79, *p*<0.001), maltose (*χ*^2^=33.85, *p*<0.001). However, success after feeding on artificial *L. camara* nectar was not significantly different from success after feeding on sucrose alone (*χ*^2^=0.18, *p*=0.673) or fructose alone (*χ*^2^=1.41, *p*=0.235).

### 4.6 Single-sugar solutions compared

When the solutions were 20% sugar, prey-capture success was significantly higher after feeding on sucrose than after feeding on glucose (*χ*^2^=13.32, *p*<0.001; figure 3*a*,*c*) or maltose (*χ*^2^=31.10, *p*<0.001; figure 3*a*,*d*), significantly higher after feeding on fructose than after feeding on glucose (*χ*^2^=8.93, *p*=0.003; figure 3*b*,*c*) or maltose (*χ*^2^=25.40, *p*<0.001; figure 3*b*,*d*) and also significantly higher after feeding on glucose instead of maltose (*χ*^2^=5.25, *p*=0.022; figure 3*c*,*d*). However, there was no significant difference between sucrose and fructose (*χ*^2^=0.60, *p*=0.439; figure 3*a*,*b*). When the solutions were 5% sugar, success was significantly higher after feeding on sucrose than after feeding on glucose (*χ*^2^=7.75, *p*=0.005; figure 3*a*,*c*) or maltose (*χ*^2^=17.04, *p*<0.001; figure 3*a*,*d*) and significantly higher after feeding on fructose than after feeding on glucose (*χ*^2^=10.48, *p*<0.001; figure 3*b*,*c*) or maltose (*χ*^2^=20.73, *p*<0.001; figure 3*b*,*d*), but there was no significant difference between 5% sucrose and 5% fructose (*χ*^2^=0.24, *p*=0.628; figure 3*a*,*b*), or between 5% glucose and 5% maltose (*χ*^2^=2.05, *p*=0.153; figure 3*c*,*d*). When the solutions were 1% sugar, there were no significant differences between any pairs: sucrose–fructose (*χ*^2^=0.16, *p*=0.690; figure 3*a*,*b*), sucrose–glucose (*χ*^2^=0.54, *p*=0.465; figure 3*a*,*c*), sucrose–maltose (*χ*^2^=0.77, *p*=0.379; figure 3*a*,*d*), fructose–glucose (*χ*^2^=0.19, *p*=0.659; figure 3*b*,*c*), fructose–maltose (*χ*^2^=0.35, *p*=0.556; figure 3*b*,*d*), glucose–maltose (*χ*^2^=0.02, *p*=0.882; figure 3*c*,*d*).

Spiders were significantly more successful at capturing prey after feeding on 20% sucrose (figure 3*a*) than after feeding on 5% (*χ*^2^=5.81, *p*=0.016) or 1% sucrose (*χ*^2^=37.70, *p*<0.001), and significantly more successful after feeding on 5% sucrose than after feeding on 1% sucrose (*χ*^2^=81.15, *p*<0.001). For fructose (figure 3*b*), spiders that fed from a 20% (*χ*^2^=34.57, *p*<0.001) or 5% (*χ*^2^=24.39, *p*<0.001) solution were significantly more successful than spiders that fed from a 1% solution, but there was no significant difference between 20% and 5% (*χ*^2^=1.4, *p*=0.226). For glucose (figure 3*c*), spiders were significantly more successful after feeding from a 20% (*χ*^2^=13.53, *p*<0.001) or a 5% (*χ*^2^=5.25, *p*=0.022) solution than after feeding from a 1% solution, but there was no significant difference between 20% and 5% glucose (*χ*^2^=2.10, *p*=0.148). When the sugar was maltose (figure 3*d*), there were no significant differences when concentrations were compared: 20–5% (*χ*^2^=0.33, *p*=0.565), 20–1% (*χ*^2^=2.54, *p*=0.111), 5–1% (*χ*^2^=1.05, *p*=0.306).

### 4.7 Plants and single-sugar solutions compared

The prey-capture success of spiders that fed on *L. camara* was not significantly different from the success of spiders that fed on either 20% sucrose (*χ*^2^=0.41, *p*=0.52; figures 2*a* and 3*a*) or 20% fructose (*χ*^2^=1.97, *p*=0.161; figures 2*a* and 3*b*). Prey-capture success of spiders that fed on 20% sucrose was significantly higher than the success of spiders that fed on *R. communis* (*χ*^2^=5.81, *p*=0.016; figures 2*a* and 3*a*), although there was no significant difference between *R. communis* and 20% fructose (*χ*^2^=2.84, *p*=0.092; figures 2*a* and 3*b*). The success of spiders that fed on *P. hysterophorus* was significantly lower than the success of spiders that fed on either 20% sucrose (*χ*^2^=23.82, *p*<0.001; figures 2*a* and 3*a*) or 20% fructose (*χ*^2^=18.46, *p*<0.001; figures 2*a* and 3*b*).

## 5. Discussion

Numerous studies have shown that sugars and amino acids acquired by feeding on nectar can have beneficial effects on the growth, survival and reproduction of insects (e.g. [56–59]), but our objective was different. We investigated rapidly expressed benefits that apply during a particular phase in a spider's life, namely the phase immediately after the spider emerges from egg sacs and before it has its first prey meal. The specific benefit we considered was plant meal derived improvement in prey-capture proficiency 1 day after the meal and the plant species we considered were *L. camara*, *R. communis* and *P. hysterophorus*. As predicted, we found that, compared with spiders from the water-only control, spiders that fed on these plants were significantly more successful at capturing prey.

The particular plant species from which the juvenile acquired its nectar meal also mattered. In our experiments, and in an earlier study [46], we never saw a spider enter or bite into flowers and instead we saw spiders feed by pressing their mouthparts against petals, leaves and stems of the plant and, when the plant was *R. communis*, drops of nectar from EFNs. Conspicuous EFNs are characteristic of *R. communis* [60,61], but not characteristic of *L. camara* or *P. hysterophorus*. However, many plants have EFNs and EFNs are not always conspicuous [62]. Even *R. communis* has, besides its large, conspicuous EFNs, additional EFNs that are evident only with magnification [63].

It is unlikely that the spider fed on phloem or plant tissue instead of nectar. Although nectar is derived primarily from phloem, fructose is characteristic of nectar, whereas phloem is dominated by sucrose alone [64]. Moreover, cold-anthrone testing from an earlier study [46] confirmed that *E. culicivora* ingests fructose when pressing its mouthparts against the surface of the three plant species we used.

It has been suggested that the volume of nectar provided by *P. hysterophorus* is especially low [65], and yet cold-anthrone testing showed that *E. culicivora* acquires fructose from this plant [46] and we have now shown that, after feeding on *P. hysterophorus*, spiders become significantly more successful than the control spiders at capturing prey, but not as successful as spiders that fed on *L. camara*. If nectar volume matters, then spiders from the *L. camara* group being significantly more successful than spiders from the *P. hysterophorus* group is as expected [65]. However, spiders from the *L. camara* group were also significantly more successful than spiders from the *R. communis* group, despite the copious secretion of nectar from EFNs being characteristic of *R. communis*. These findings suggest that, for the plant species we used, the primary influence on prey-capture success is something other than simply variation in the nectar volume available to *E. culicivora*.

Our findings also suggest that the presence of amino acids (or at least the four dominant amino acids) in nectar was not a primary influence on prey-capture success, but that the particular sugars present in a solution, and their concentrations, did matter. The prey-capture success of spiders from each of our 1% single-sugar groups (sucrose, fructose, glucose and maltose) was not significantly different from the success of spiders from the water-only control, nor were there any significant differences between spiders that fed on the different sugars at 1%. However, findings from using 5% and 20% solutions revealed a ranking of the four sugars: maltose lowest, glucose intermediate, sucrose and fructose tied for highest.

Spiders that fed on sucrose and fructose at concentrations of 5% or 20% were significantly more successful than spiders that fed on glucose or maltose at the same concentrations. We also found that, when the single-sugar concentration was 5% or 20%, spiders that fed on sucrose and spiders that fed on fructose became significantly more successful than spiders from the water-only control, but there was no significant difference between the sucrose and fructose groups when concentration was 5% or 20%.

A combination of findings implies that glucose was intermediate between maltose and sucrose–fructose. Spiders that fed on 20% glucose alone were significantly less successful than spiders that fed on 20% sucrose or 20% fructose, but they were significantly more successful than spiders that fed on 20% maltose or spiders from the water-only control. However, the success of spiders that fed on 5% glucose, although significantly better than the success of spiders from the control group, was not significantly different from the success of spiders that fed on 5% maltose.

Fructose and glucose are monosaccharides, but sucrose and maltose are disaccharides. The hydrolysis of sucrose releases fructose and glucose, but maltose hydrolysis releases only glucose. Spiders from the 20% glucose group were significantly more successful than spiders from the 20% maltose group and, at all concentrations, the success of spiders that fed on maltose alone was not significantly different from the success of spiders from the water-only control. This combination of findings suggests that the spider has little capacity for maltose hydrolysis and also suggests that acquiring glucose in addition to fructose from sucrose is of little or no advantage over solely acquiring the fructose (i.e. there was no significant difference between 20% sucrose group and the 20% fructose group).

Sucrose, fructose and glucose are known to be present in roughly comparable ratios in the floral nectar of *L. camara* (Irene Baker cited in [51]) and the EFN of *R. communis* [60], and the same sugars in similar ratios might be expected for the nectar of *P. hysterophorus* [49,52]. The explanation for *L. camara* being ranked best, *R. communis* intermediate and *P. hysterophorus* worst might have more to do with interspecific variation in sugar concentration instead of interspecific variation in the ratios of the available sugars, but testing this hypothesis will probably be especially difficult. The concentration of sugar in nectar is known to be sensitive to relative humidity, time of day and other environmental factors [66–68], and estimating the sugar concentration encountered by a spider when it presses its mouthparts on the surfaces of the different plant species might be especially difficult. However, we can propose how the spider's access to sucrose and especially fructose might vary across the plant species we used in our experiments.

Finding no significant difference between the success of spiders that fed on 20% sucrose or 20% fructose and the success of spiders that fed on *L. camara* suggests that, on *L. camara*, *E. culicivora* juveniles can gain access to one or both of these sugars at an optimal concentration. That the spiders we let feed on 20% sucrose or 20% fructose were significantly more successful at prey-capture than the spiders we let feed on *R. communis* or *P. hysterophorus* suggests that, on these two plant species, *E. culicivora* juveniles cannot readily gain access to either of these sugars at the concentration available from *L. camara*. However, there are alternative hypotheses we cannot rule out at this stage. For example, we cannot rule out a hypothesis that unknown non-sugar compounds from *R. communis* and *P. hysterophorus*, but not *L. camara*, had negative effects on prey-capture success [69].

Spiders were less successful at capturing prey after a 6 day fast than after a 3 day fast, suggesting that longer fasting weakened the spider. Yet, the distribution of success rates across groups followed much the same pattern irrespective of fasting duration. These findings suggest that, although hungrier spiders benefit more from plant-derived nutrients, the benefit-related ranking of the nutrient sources is stable across hunger level.

Nectar and other plant-derived nutrients may often be important in the natural diets of spiders and predatory insects and, when the predators kill agricultural pests, there is an impetus to determine whether ensuring the availability of plant meal sources might make predators more effective in the biological control of the pest species [70,71]. When discussing agricultural systems, the most frequently considered beneficial effects of nectar meals include the sustaining of predator populations during periods of prey scarcity, giving predators access to nutrients not available from prey and reducing the level of competition between predators that target the same prey (e.g. [72]). These benefits would normally be expressed over a considerable timespan and comparable long-term benefits may apply to *E. culicivora*. However, the benefits implied by our findings are expressed the next day after a nectar meal and it might be of interest to investigate whether similar rapid benefits apply to other predators, including predators that target agricultural pests.

## Supplementary Material

Supplementary Data File This file can be used as a Supplementary File or it can be loaded later into Dryad. I do not understand how Dryad works. If it is easier just to use this as Supplementary, then that is OK with me.

## Data accessibility

Data available from the Dryad digital repository: http://dx.doi.org/10.5061/dryad ().
