# Supplementary material for: Rapid nectar-meal effects on a predator's capacity to kill mosquitoes
Source: R Soc Open Sci. 2015 May 13;2(5):140426. doi: 10.1098/rsos.140426 (PMC4453243; doi:10.1098/rsos.140426)
Supplement: Supplementary Data File This file can be used as a Supplementary File or it can be loaded later into Dryad. I do not understand how Dryad works. If it is easier just to use this as Supplementary, then that is OK with me. [file rsos140426supp1.doc]

RRJ.Raw Data.1st-meal effects.30mar15

We assigned spiders at random to one of 18 meal-type groups and, depending on the group, gave the spider access to a first meal on a specified day. For each water-only control group, the individuals used were derived from at 29 sibships. For each other group, the individuals used were derived from at 8-10 sibships. A ‘sibship’ is defined as the progeny of a particular male and female. No sibships contributed individuals to more than one group. The number of individuals from any one sibship was never more than eight or less than three. As the number of sibships per group was large and the number of individuals from each sibship was small, we did not include sibship as a variable in our data analyses.

The first meal was artificial nectar (i.e., a solution of sugar or sugar plus amino acids), a plant or a water-only control (table 1). The plants were *L*. *camara* and *R*. *communis*, and also *Parthenium hysterophorus*, another species that is common in the same habitat but not known to attract *E*. *culicivora* in olfactometer experiments (unpubl. data).

The sugar and amino acid content of *L*. *camara* nectar is known (Irene Baker pers. comm., cited by [50]): sucrose 187.25 g/L, fructose 57.00 g/L, glucose 55.80 g/L, proline 0.256 g/L, glycine 0.178 g/L, serine 0.144 g/L, glutamine 0.136 g/L, threonine 0.080 g/L, alanine 0.064 g/L, asparagine 0.056 g/L, tyrosine 0.040 g/L, glutamic acid 0.048 g/L, arginine 0.032 g/L, valine 0.016 g/L. For our experiments, we made two artificial nectar blends based on the reported ratio of compounds (the three sugars and the four dominant amino acids) in this plant’s nectar.

Full artificial *L*. *camara* nectar: sucrose 187.3 g/L, fructose 57.0 g/L, glucose 55.8 g/L, proline 0.3 g/L, glycine 0.2 g/L, serine 0.1 g/L, glutamine 0.1 g/L.

Sugar-only artificial *L*. *camara* nectar: sucrose 187.3 g/L, fructose 57.0 g/L, glucose 55.8 g/L.

The nectar-sugar content of *R*. *communis* and *P*. *hysterophorus* is unknown, but the floral tissues of these plants contain sucrose, fructose, glucose and other sugars, including especially maltose [51], which we include in our experiments as a sugar that is not known to be present in the nectar of *L*. *camara* or prevalent in the nectar of plants in general.

For each meal-type group, there were two fast-duration subgroups (3-day and 6-day): spiders kept without access to food for 3 days or 6 days before being given access to the meal corresponding to the meal type group (table 1) on the 4^th^ or 7^th^ day. For each group (row in the table), N = 50 successful tests, except that N = 200 for each control (distilled water on day 4 and on day 5). ‘Successful means the spider attacked a prey individual before 120 min elapsed. Outcome: captured prey (i.e., the spider held on to prey, prey became inactive and spider fed) or did not capture prey (i.e. spider failed to hold on – it was shaken off by the prey). Prey-capture proficiency: percentage of N spiders that captured prey.

| Group | Meal-type group | Dilution | Fast duration (F) in days | Sibship | N | Captured prey |
| --- | --- | --- | --- | --- | --- | --- |
| LC-C (F3) | *Lantana camara* cutting | NA | 3 | 1 | 6 | 6 |
|  |  |  |  | 2 | 6 | 6 |
|  |  |  |  | 3 | 6 | 6 |
|  |  |  |  | 4 | 6 | 6 |
|  |  |  |  | 5 | 5 | 5 |
|  |  |  |  | 6 | 5 | 4 |
|  |  |  |  | 7 | 4 | 4 |
|  |  |  |  | 8 | 4 | 4 |
|  |  |  |  | 9 | 4 | 4 |
|  |  |  |  | 10 | 4 | 3 |
|  |  |  |  | Total | 50 | 48 (96%) |
| LC-C (F6) | *Lantana* *camara* cutting | NA | 6 | 1 | 6 | 6 |
|  |  |  |  | 2 | 6 | 5 |
|  |  |  |  | 3 | 6 | 5 |
|  |  |  |  | 4 | 6 | 5 |
|  |  |  |  | 5 | 6 | 3 |
|  |  |  |  | 6 | 5 | 5 |
|  |  |  |  | 7 | 5 | 5 |
|  |  |  |  | 8 | 5 | 4 |
|  |  |  |  | 9 | 5 | 3 |
|  |  |  |  | Total | 50 | 41 (82%) |
| RC-C (F3) | *Ricinus communis cutting* | NA | 3 | 1 | 7 | 6 |
|  |  |  |  | 2 | 6 | 6 |
|  |  |  |  | 3 | 6 | 5 |
|  |  |  |  | 4 | 6 | 3 |
|  |  |  |  | 5 | 6 | 3 |
|  |  |  |  | 6 | 5 | 5 |
|  |  |  |  | 7 | 5 | 4 |
|  |  |  |  | 8 | 5 | 4 |
|  |  |  |  | 9 | 4 | 3 |
|  |  |  |  | Total | 50 | 39 (78%) |
| RC-C (F6) | *Ricinus communis* cutting | NA | 6 | 1 | 6 | 5 |
|  |  |  |  | 2 | 6 | 4 |
|  |  |  |  | 3 | 6 | 4 |
|  |  |  |  | 4 | 6 | 3 |
|  |  |  |  | 5 | 5 | 4 |
|  |  |  |  | 6 | 5 | 3 |
|  |  |  |  | 7 | 4 | 3 |
|  |  |  |  | 8 | 4 | 3 |
|  |  |  |  | 9 | 4 | 2 |
|  |  |  |  | 10 | 4 | 2 |
|  |  |  |  | Total | 50 | 33 (66%) |
| PH-C (F3) | *Parthenium hysterophorus cutting* | NA | 3 | 1 | 7 | 4 |
|  |  |  |  | 2 | 6 | 5 |
|  |  |  |  | 3 | 6 | 4 |
|  |  |  |  | 4 | 6 | 3 |
|  |  |  |  | 5 | 5 | 4 |
|  |  |  |  | 6 | 5 | 4 |
|  |  |  |  | 7 | 5 | 4 |
|  |  |  |  | 8 | 5 | 3 |
|  |  |  |  | 9 | 5 | 3 |
|  |  |  |  | Total | 50 | 34 (68%) |
| PH-C (F6) | *Parthenium hysterophorus* cutting | NA | 6 | 1 | 6 | 3 |
|  |  |  |  | 2 | 6 | 3 |
|  |  |  |  | 3 | 6 | 2 |
|  |  |  |  | 4 | 6 | 2 |
|  |  |  |  | 5 | 6 | 2 |
|  |  |  |  | 6 | 5 | 3 |
|  |  |  |  | 7 | 5 | 2 |
|  |  |  |  | 8 | 5 | 1 |
|  |  |  |  | 9 | 5 | 1 |
|  |  |  |  | Total | 50 | 19 (38%) |
| LC-SAA (F3) | Full artificial nectar of *L*. *camara* | * | 3 | 1 | 7 | 6 |
|  |  |  |  | 2 | 6 | 6 |
|  |  |  |  | 3 | 6 | 4 |
|  |  |  |  | 4 | 5 | 5 |
|  |  |  |  | 5 | 5 | 5 |
|  |  |  |  | 6 | 5 | 5 |
|  |  |  |  | 7 | 4 | 4 |
|  |  |  |  | 8 | 4 | 4 |
|  |  |  |  | 9 | 4 | 4 |
|  |  |  |  | 10 | 4 | 2 |
|  |  |  |  | Total | 50 | 45 (90%) |
| LC-SAA (F6) | Full artificial nectar of *L*. *camara* | * | 6 | 1 | 7 | 6 |
|  |  |  |  | 2 | 7 | 5 |
|  |  |  |  | 3 | 6 | 6 |
|  |  |  |  | 4 | 6 | 4 |
|  |  |  |  | 5 | 5 | 5 |
|  |  |  |  | 6 | 5 | 5 |
|  |  |  |  | 7 | 5 | 3 |
|  |  |  |  | 8 | 5 | 3 |
|  |  |  |  | 9 | 4 | 3 |
|  |  |  |  | Total | 50 | 40 (80%) |
| LC-S (F3) | *Sugar-only artificial nectar of L. camara* | ** | 3 | 1 | 6 | 6 |
|  |  |  |  | 2 | 6 | 6 |
|  |  |  |  | 3 | 6 | 5 |
|  |  |  |  | 4 | 5 | 5 |
|  |  |  |  | 5 | 5 | 5 |
|  |  |  |  | 6 | 5 | 5 |
|  |  |  |  | 7 | 5 | 5 |
|  |  |  |  | 8 | 4 | 4 |
|  |  |  |  | 9 | 4 | 4 |
|  |  |  |  | 10 | 4 | 2 |
|  |  |  |  | Total | 50 | 47 (94%) |
| LC-S (F6) | *Sugar-only artificial nectar of L. camara* | ** | 6 | 1 | 7 | 6 |
|  |  |  |  | 2 | 7 | 6 |
|  |  |  |  | 3 | 6 | 5 |
|  |  |  |  | 4 | 6 | 5 |
|  |  |  |  | 5 | 5 | 5 |
|  |  |  |  | 6 | 5 | 5 |
|  |  |  |  | 7 | 5 | 4 |
|  |  |  |  | 8 | 5 | 3 |
|  |  |  |  | 9 | 4 | 2 |
|  |  |  |  | Total | 50 | 41 (82%) |
| Suc-20 (F3) | Sucrose-only artificial nectar at high concentration (20%) | 20% | 3 | 1 | 7 | 7 |
|  |  |  |  | 2 | 7 | 6 |
|  |  |  |  | 3 | 6 | 5 |
|  |  |  |  | 4 | 5 | 5 |
|  |  |  |  | 5 | 5 | 5 |
|  |  |  |  | 6 | 5 | 5 |
|  |  |  |  | 7 | 5 | 5 |
|  |  |  |  | 8 | 5 | 4 |
|  |  |  |  | 9 | 5 | 4 |
|  |  |  |  | Total | 50 | 46 (92%) |
| Suc-20 (F6) | Sucrose-only artificial nectar at high concentration (20%) | 20% | 6 | 1 | 7 | 5 |
|  |  |  |  | 2 | 6 | 6 |
|  |  |  |  | 3 | 6 | 6 |
|  |  |  |  | 4 | 6 | 5 |
|  |  |  |  | 5 | 6 | 5 |
|  |  |  |  | 6 | 5 | 3 |
|  |  |  |  | 7 | 5 | 3 |
|  |  |  |  | 8 | 3 | 3 |
|  |  |  |  | 9 | 3 | 3 |
|  |  |  |  | 10 | 3 | 1 |
|  |  |  |  | Total | 50 | 40 (80%) |
| Suc-5 (F3 | Sucrose-only artificial nectar at medium concentration (5%) | 5% | 3 | 1 | 8 | 6 |
|  |  |  |  | 2 | 6 | 6 |
|  |  |  |  | 3 | 6 | 5 |
|  |  |  |  | 4 | 6 | 5 |
|  |  |  |  | 5 | 6 | 5 |
|  |  |  |  | 6 | 6 | 4 |
|  |  |  |  | 7 | 6 | 4 |
|  |  |  |  | 8 | 6 | 4 |
|  |  |  |  | Total | 50 | 39 (78%) |
| Suc-5 (F6) | Sucrose-only artificial nectar at medium concentration (5%) | 5% | 6 | 1 | 7 | 5 |
|  |  |  |  | 2 | 7 | 5 |
|  |  |  |  | 3 | 6 | 4 |
|  |  |  |  | 4 | 5 | 4 |
|  |  |  |  | 5 | 5 | 3 |
|  |  |  |  | 6 | 5 | 3 |
|  |  |  |  | 7 | 5 | 3 |
|  |  |  |  | 8 | 5 | 3 |
|  |  |  |  | 9 | 5 | 3 |
|  |  |  |  | Total | 50 | 33 (66%) |
| Suc-1 (F3) | Sucrose-only artificial nectar at low concentration (1%) | 1% | 3 | 1 | 6 | 4 |
|  |  |  |  | 2 | 6 | 3 |
|  |  |  |  | 3 | 6 | 3 |
|  |  |  |  | 4 | 6 | 2 |
|  |  |  |  | 5 | 6 | 2 |
|  |  |  |  | 6 | 6 | 2 |
|  |  |  |  | 7 | 4 | 3 |
|  |  |  |  | 8 | 4 | 2 |
|  |  |  |  | 9 | 3 | 1 |
|  |  |  |  | 10 | 3 | 1 |
|  |  |  |  | Total | 50 | 23 (46%) |
| Suc-1 (F6) | Sucrose-only artificial nectar at low concentration (1%) | 1% | 6 | 1 | 6 | 4 |
|  |  |  |  | 2 | 6 | 2 |
|  |  |  |  | 3 | 5 | 4 |
|  |  |  |  | 4 | 5 | 2 |
|  |  |  |  | 5 | 5 | 1 |
|  |  |  |  | 6 | 5 | 1 |
|  |  |  |  | 7 | 5 | 1 |
|  |  |  |  | 8 | 5 | 1 |
|  |  |  |  | 9 | 4 | 2 |
|  |  |  |  | 10 | 4 | 1 |
|  |  |  |  | Total | 50 | 19 (38%) |
| Fru-20 (F3) | Fructose-only artificial nectar at high concentration (20%) | 20% | 3 | 1 | 7 | 7 |
|  |  |  |  | 2 | 7 | 6 |
|  |  |  |  | 3 | 7 | 6 |
|  |  |  |  | 4 | 7 | 6 |
|  |  |  |  | 5 | 6 | 6 |
|  |  |  |  | 6 | 6 | 5 |
|  |  |  |  | 7 | 5 | 5 |
|  |  |  |  | 8 | 5 | 4 |
|  |  |  |  | Total | 50 | 45 (90%) |
| Fru-20 (F6) | Fructose-only artificial nectar at high concentration (20%) | 20% | 6 | 1 | 7 | 5 |
|  |  |  |  | 2 | 5 | 5 |
|  |  |  |  | 3 | 5 | 4 |
|  |  |  |  | 4 | 5 | 4 |
|  |  |  |  | 5 | 5 | 4 |
|  |  |  |  | 6 | 5 | 4 |
|  |  |  |  | 7 | 5 | 4 |
|  |  |  |  | 8 | 5 | 2 |
|  |  |  |  | 9 | 4 | 3 |
|  |  |  |  | 10 | 4 | 2 |
|  |  |  |  | Total | 50 | 37 (74%) |
| Fru-5 (F3) | Fructose-only artificial nectar at medium concentration (5%) | 5% | 3 | 1 | 6 | 6 |
|  |  |  |  | 2 | 6 | 6 |
|  |  |  |  | 3 | 6 | 5 |
|  |  |  |  | 4 | 6 | 5 |
|  |  |  |  | 5 | 6 | 4 |
|  |  |  |  | 6 | 5 | 5 |
|  |  |  |  | 7 | 4 | 4 |
|  |  |  |  | 8 | 4 | 2 |
|  |  |  |  | 9 | 4 | 3 |
|  |  |  |  | 10 | 3 | 2 |
|  |  |  |  | Total | 50 | 42 (84%) |
| Fru-5 (F6) | Fructose-only artificial nectar at medium concentration (5%) | 5% | 6 | 1 | 6 | 6 |
|  |  |  |  | 2 | 6 | 4 |
|  |  |  |  | 3 | 6 | 4 |
|  |  |  |  | 4 | 6 | 4 |
|  |  |  |  | 5 | 6 | 4 |
|  |  |  |  | 6 | 6 | 4 |
|  |  |  |  | 7 | 6 | 1 |
|  |  |  |  | 8 | 4 | 4 |
|  |  |  |  | 9 | 4 | 2 |
|  |  |  |  | Total | 50 | 33 (66%) |
| Fru-1 (F3) | Fructose-only artificial nectar at low concentration (1%) | 1% | 3 | 1 | 8 | 3 |
|  |  |  |  | 2 | 6 | 5 |
|  |  |  |  | 3 | 6 | 2 |
|  |  |  |  | 4 | 5 | 3 |
|  |  |  |  | 5 | 5 | 2 |
|  |  |  |  | 6 | 5 | 3 |
|  |  |  |  | 7 | 5 | 2 |
|  |  |  |  | 8 | 5 | 1 |
|  |  |  |  | 9 | 5 | 1 |
|  |  |  |  | Total | 50 | 22 (44%) |
| Fru-1 (F6) | Fructose-only artificial nectar at low concentration (1%) | 1% | 6 | 1 | 7 | 4 |
|  |  |  |  | 2 | 7 | 2 |
|  |  |  |  | 3 | 6 | 2 |
|  |  |  |  | 4 | 5 | 1 |
|  |  |  |  | 5 | 5 | 1 |
|  |  |  |  | 6 | 4 | 3 |
|  |  |  |  | 7 | 4 | 2 |
|  |  |  |  | 8 | 4 | 1 |
|  |  |  |  | 9 | 4 | 1 |
|  |  |  |  | 10 | 4 | 1 |
|  |  |  |  | Total | 50 | 18 (36%) |
| Glu-20 (F3) | Glucose-only artificial nectar at high concentration (20%) | 20% | 3 | 1 | 7 | 6 |
|  |  |  |  | 2 | 7 | 5 |
|  |  |  |  | 3 | 6 | 5 |
|  |  |  |  | 4 | 6 | 5 |
|  |  |  |  | 5 | 6 | 5 |
|  |  |  |  | 6 | 6 | 4 |
|  |  |  |  | 7 | 6 | 4 |
|  |  |  |  | 8 | 6 | 3 |
|  |  |  |  | Total | 50 | 37 (74%) |
| Glu-20 (F6) | Glucose-only artificial nectar at high concentration (20%) | 20% | 6 | 1 | 7 | 3 |
|  |  |  |  | 2 | 7 | 3 |
|  |  |  |  | 3 | 5 | 4 |
|  |  |  |  | 4 | 5 | 4 |
|  |  |  |  | 5 | 5 | 4 |
|  |  |  |  | 6 | 5 | 4 |
|  |  |  |  | 7 | 5 | 1 |
|  |  |  |  | 8 | 5 | 1 |
|  |  |  |  | 9 | 3 | 1 |
|  |  |  |  | 10 | 3 | 1 |
|  |  |  |  | Total | 50 | 26 (52%) |
| Glu-5 (F3) | Glucose-only artificial nectar at medium concentration (5%) | 5% | 3 | 1 | 6 | 5 |
|  |  |  |  | 2 | 6 | 4 |
|  |  |  |  | 3 | 6 | 3 |
|  |  |  |  | 4 | 6 | 3 |
|  |  |  |  | 5 | 6 | 3 |
|  |  |  |  | 6 | 6 | 3 |
|  |  |  |  | 7 | 4 | 3 |
|  |  |  |  | 8 | 4 | 2 |
|  |  |  |  | 9 | 3 | 3 |
|  |  |  |  | 10 | 3 | 1 |
|  |  |  |  | Total | 50 | 30 (60%) |
| Glu-5 (F6) | Glucose-only artificial nectar at medium concentration (5%) | 5% | 6 | 1 | 8 | 3 |
|  |  |  |  | 2 | 6 | 4 |
|  |  |  |  | 3 | 6 | 4 |
|  |  |  |  | 4 | 6 | 3 |
|  |  |  |  | 5 | 6 | 3 |
|  |  |  |  | 6 | 6 | 2 |
|  |  |  |  | 7 | 6 | 2 |
|  |  |  |  | 8 | 6 | 2 |
|  |  |  |  | Total | 50 | 23 (46%) |
| Glu-1 (F3) | Glucose-only solution | 1% | 3 | 1 | 8 | 3 |
|  |  |  |  | 2 | 7 | 3 |
|  |  |  |  | 3 | 6 | 3 |
|  |  |  |  | 4 | 6 | 3 |
|  |  |  |  | 5 | 6 | 3 |
|  |  |  |  | 6 | 6 | 3 |
|  |  |  |  | 7 | 6 | 1 |
|  |  |  |  | 8 | 5 | 1 |
|  |  |  |  | Total | 50 | 20 (40%) |
| Glu-1 (F6) | Glucose-only artificial nectar at low concentration (1%) | 1% | 6 | 1 | 6 | 3 |
|  |  |  |  | 2 | 6 | 2 |
|  |  |  |  | 3 | 6 | 2 |
|  |  |  |  | 4 | 6 | 1 |
|  |  |  |  | 5 | 5 | 2 |
|  |  |  |  | 6 | 5 | 1 |
|  |  |  |  | 7 | 4 | 3 |
|  |  |  |  | 8 | 4 | 1 |
|  |  |  |  | 9 | 4 | 1 |
|  |  |  |  | 10 | 4 | 1 |
|  |  |  |  | Total | 50 | 17 (34%) |
| Mal-20 (F3) | Maltose-only solution | 20% | 3 | 1 | 6 | 4 |
|  |  |  |  | 2 | 6 | 4 |
|  |  |  |  | 3 | 6 | 3 |
|  |  |  |  | 4 | 6 | 2 |
|  |  |  |  | 5 | 6 | 2 |
|  |  |  |  | 6 | 4 | 4 |
|  |  |  |  | 7 | 4 | 3 |
|  |  |  |  | 8 | 4 | 2 |
|  |  |  |  | 9 | 4 | 2 |
|  |  |  |  | 10 | 4 | 1 |
|  |  |  |  | Total | 50 | 27 (54%) |
| Mal-20 (F6) | Maltose-only artificial nectar at high concentration (20%) | 20% | 6 | 1 | 6 | 4 |
|  |  |  |  | 2 | 6 | 3 |
|  |  |  |  | 3 | 6 | 2 |
|  |  |  |  | 4 | 6 | 2 |
|  |  |  |  | 5 | 6 | 2 |
|  |  |  |  | 6 | 6 | 2 |
|  |  |  |  | 7 | 4 | 2 |
|  |  |  |  | 8 | 4 | 1 |
|  |  |  |  | 9 | 3 | 1 |
|  |  |  |  | 10 | 3 | 1 |
|  |  |  |  | Total | 50 | 20 (40%) |
| M5 (F3) | Maltose-only artificial nectar at high concentration (20%) | 5% | 3 | 1 | 6 | 3 |
|  |  |  |  | 2 | 6 | 2 |
|  |  |  |  | 3 | 5 | 4 |
|  |  |  |  | 4 | 5 | 3 |
|  |  |  |  | 5 | 5 | 3 |
|  |  |  |  | 6 | 5 | 2 |
|  |  |  |  | 7 | 5 | 2 |
|  |  |  |  | 8 | 5 | 2 |
|  |  |  |  | 9 | 4 | 2 |
|  |  |  |  | 10 | 4 | 2 |
|  |  |  |  | Total | 50 | 25 (50%) |
| Mal-5 (F6) | Maltose-only artificial nectar at medium concentration (5%) | 5% | 6 | 1 | 7 | 2 |
|  |  |  |  | 2 | 6 | 4 |
|  |  |  |  | 3 | 6 | 3 |
|  |  |  |  | 4 | 6 | 2 |
|  |  |  |  | 5 | 6 | 2 |
|  |  |  |  | 6 | 6 | 2 |
|  |  |  |  | 7 | 5 | 1 |
|  |  |  |  | 8 | 4 | 1 |
|  |  |  |  | 9 | 4 | 1 |
|  |  |  |  | Total | 50 | 18 (36%) |
| Mal-1 (F3) | Maltose-only artificial nectar at medium concentration (1%) | 1% | 3 | 1 | 6 | 2 |
|  |  |  |  | 2 | 6 | 2 |
|  |  |  |  | 3 | 5 | 3 |
|  |  |  |  | 4 | 5 | 2 |
|  |  |  |  | 5 | 5 | 2 |
|  |  |  |  | 6 | 5 | 2 |
|  |  |  |  | 7 | 5 | 2 |
|  |  |  |  | 8 | 5 | 2 |
|  |  |  |  | 9 | 4 | 2 |
|  |  |  |  | 10 | 4 | 1 |
|  |  |  |  | Total | 50 | 20 (40%) |
| Mal-1 (F6) | Maltose-only artificial nectar at medium concentration (1%) | 1% | 6 | 1 | 6 | 3 |
|  |  |  |  | 2 | 6 | 2 |
|  |  |  |  | 3 | 6 | 2 |
|  |  |  |  | 4 | 6 | 2 |
|  |  |  |  | 5 | 6 | 2 |
|  |  |  |  | 6 | 6 | 1 |
|  |  |  |  | 7 | 6 | 1 |
|  |  |  |  | 8 | 4 | 2 |
|  |  |  |  | 9 | 4 | 1 |
|  |  |  |  | Total | 50 | 16 (32%) |
| H_2_O (F3) | Control (distilled water alone) | NA | 3 | 1 | 8 | 5 |
|  |  |  |  | 2 | 8 | 4 |
|  |  |  |  | 3 | 8 | 4 |
|  |  |  |  | 4 | 8 | 4 |
|  |  |  |  | 5 | 8 | 3 |
|  |  |  |  | 6 | 8 | 3 |
|  |  |  |  | 7 | 8 | 3 |
|  |  |  |  | 8 | 8 | 3 |
|  |  |  |  | 9 | 8 | 3 |
|  |  |  |  | 10 | 8 | 3 |
|  |  |  |  | 11 | 8 | 3 |
|  |  |  |  | 12 | 8 | 3 |
|  |  |  |  | 13 | 8 | 2 |
|  |  |  |  | 14 | 8 | 2 |
|  |  |  |  | 15 | 7 | 4 |
|  |  |  |  | 16 | 7 | 3 |
|  |  |  |  | 17 | 7 | 2 |
|  |  |  |  | 18 | 7 | 2 |
|  |  |  |  | 19 | 7 | 2 |
|  |  |  |  | 20 | 7 | 2 |
|  |  |  |  | 21 | 6 | 3 |
|  |  |  |  | 22 | 6 | 2 |
|  |  |  |  | 23 | 5 | 3 |
|  |  |  |  | 24 | 5 | 3 |
|  |  |  |  | 25 | 5 | 2 |
|  |  |  |  | 26 | 5 | 2 |
|  |  |  |  | 27 | 5 | 2 |
|  |  |  |  | 28 | 5 | 2 |
|  |  |  |  | 29 | 4 | 3 |
|  |  |  |  | Total | 200 | 82 (41%) |
| H_2_O (F6) | Control (distilled water alone) | NA | 6 | 1 | 8 | 4 |
|  |  |  |  | 2 | 8 | 3 |
|  |  |  |  | 3 | 8 | 3 |
|  |  |  |  | 4 | 8 | 3 |
|  |  |  |  | 5 | 8 | 3 |
|  |  |  |  | 6 | 8 | 2 |
|  |  |  |  | 7 | 8 | 2 |
|  |  |  |  | 8 | 8 | 2 |
|  |  |  |  | 9 | 8 | 2 |
|  |  |  |  | 10 | 7 | 3 |
|  |  |  |  | 11 | 7 | 3 |
|  |  |  |  | 12 | 7 | 3 |
|  |  |  |  | 13 | 7 | 3 |
|  |  |  |  | 14 | 7 | 2 |
|  |  |  |  | 15 | 7 | 2 |
|  |  |  |  | 16 | 7 | 2 |
|  |  |  |  | 17 | 7 | 1 |
|  |  |  |  | 18 | 6 | 3 |
|  |  |  |  | 19 | 6 | 3 |
|  |  |  |  | 20 | 6 | 2 |
|  |  |  |  | 21 | 6 | 2 |
|  |  |  |  | 22 | 6 | 2 |
|  |  |  |  | 23 | 6 | 2 |
|  |  |  |  | 24 | 6 | 2 |
|  |  |  |  | 25 | 6 | 1 |
|  |  |  |  | 26 | 6 | 1 |
|  |  |  |  | 27 | 6 | 1 |
|  |  |  |  | 28 | 6 | 1 |
|  |  |  |  | 29 | 6 | 1 |
|  |  |  |  | Total | 200 | 64 (32%) |

NA: not applicable

*Synthetic *Lantana* nectar: approximates nectar of *Lantana* *camara* (known sugars + available amino acids, in ratios and dilutions reported for L. camara nectar)

***Lantana* 3-sugar solution (ratio & dilution approximates that of *L*. *camara* nectar; no amino acids)
